# Brief Psychiatric Rating Scale – Expanded version: Construct validity using Rasch model analysis

**DOI:** 10.4102/sajpsychiatry.v31i0.2343

**Published:** 2025-05-15

**Authors:** Ashleigh J. Alford, Daleen Casteleijn, Lesley J. Robertson

**Affiliations:** 1 Department of Psychiatry, Faculty of Health Sciences, University of the Witwatersrand, Johannesburg, South Africa; 2 Department of Occupational Therapy, Faculty of Health Sciences, University of Pretoria, Pretoria, South Africa

**Keywords:** BPRS-E, construct validity, community psychiatry, Rasch model analysis, South Africa

## Abstract

**Background:**

The Brief Psychiatric Rating Scale – Expanded version (BPRS-E) is a 24-item clinician-administered scale whereby severity of psychopathology is rated using seven scoring categories for each item. Although useful in research and clinical settings, the construct validity has not been tested in South Africa.

**Aim:**

Examine the construct validity of the BPRS-E using Rasch model analysis.

**Setting:**

Community psychiatric clinics in the Sedibeng District of Gauteng province, South Africa.

**Methods:**

A retrospective record review was conducted of adult psychiatric patients in whom the BPRS-E was used in routine clinical assessment by trained psychiatric nurses and doctors. Clinical records with completed BPRS-Es were purposively sampled from three community psychiatric clinics in the Sedibeng District. Data were entered into RUMM2030^®^ software, and construct validity was analysed using the Rasch model, a probabilistic model that assesses item fit, response category functioning, and unidimensionality.

**Results:**

Clinical records of 192 patients (93 males and 99 females; aged between 18 and 79 years) were reviewed. Total BPRS-E scores ranged from 24 to 93, with a score of 39 or less in 52% of records (*n* = 100). Rasch analysis revealed good item fit and unidimensionality for the 24 BPRS-E items but disorganised threshold curves and inconsistent differential item functioning for the severity scoring categories.

**Conclusion:**

This study supports the construct validity of the BPRS-E items when used clinically in a South African community psychiatric setting. However, severity scoring using the BPRS-E scoring categories in this setting requires further investigation.

**Contribution:**

This study provides evidence that the BPRS-E is valid in a community psychiatric setting in South Africa.

## Introduction

In mental health care, monitoring of target symptoms has been shown to improve the effectiveness of interventions, prevent relapse and improve therapeutic outcomes.^1^ Additionally, worsening or persistence of symptoms is associated with reduced treatment adherence.^2^ Monitoring the severity of psychopathology over time is not only useful for adjusting treatment but may assist in improving quality of life and treatment adherence as well.^2,3^

The Brief Psychiatric Rating Scale – Expanded Version (BPRS-E) provides an assessment of common psychiatric symptoms and signs, facilitating assessment of treatment response.^1,4^ The BPRS-E is a 24-item ordinal scale that allows the clinician to rate an individual’s response as guided by semi-structured questions (which may be adapted to culture and/or language) as well as considering collateral information and clinical observation. Each item is rated on a 7-point Likert-type scale, where 1 = not present and 7 = extremely severe.^4^ Whilst the total score can theoretically range from 24 to 168, it is not possible to score maximally on all items, as some symptoms and signs are mutually exclusive (e.g. motor retardation vs. motor hyperactivity). If marked lability is present, such as in a mixed mood episode, the various symptom clusters are scored but are not all scored maximally, as this would be contradictory. The scoring categories for severity are fixed and based on the frequency and intensity of symptoms and behaviour.

Tools used to monitor symptom severity should be able to assess multiple symptom clusters to give an accurate account of psychopathology. The BPRS-E factor structure is considered to reflect the major dimensions of psychopathology and is especially relevant considering the current move towards more dimensional diagnostic models.^5^

The BPRS-E may, therefore, provide intervention opportunities within a community outpatient setting in the form of secondary and tertiary prevention. In secondary prevention, it may assist in detecting early changes in symptom severity and providing the opportunity to slow or ameliorate disease progress. In tertiary prevention, where psychiatric illness has resulted in established symptomatology, monitoring severity in response to biopsychosocial interventions may assist in lessening the impact of the illness on quality of life, level of functioning and life expectancy. A cross-sectional study in the Sedibeng District of Gauteng province, South Africa, found that residual psychiatric symptoms, measured using the BPRS-E, predicted poor perceived quality of life.^6^

## Pilot in Sedibeng District mental health services

Sedibeng is a peri-urban district municipality of Gauteng province in South Africa, covering an area of 4100 km^2^.^7^ The district mental health services are comprised of primary mental health care and community psychiatry, whereby outpatient psychiatric care is provided to community-dwelling people with severe mental illness from nine primary health care clinics.^6,8^

As of the beginning of 2019, the BPRS-E was incorporated into routine nurse and doctor assessments of people attending the community psychiatric clinics to monitor symptom severity and treatment response. Prior to implementation, the head of Sedibeng community psychiatry (LR) conducted training of staff (psychiatric consultants, registrars, medical officers and psychiatric nurses), using the BPRS-E administration manual,^4^ in 2-h weekly sessions over 6 weeks. Whilst repeated training sessions and discussions were held, the validity and inter-rater reliability of the BPRS-E were not tested.

To our knowledge, Sedibeng District’s routine use of the BPRS-E is the first of its kind in South Africa. There is thus a need for greater understanding of the applicability of the BPRS-E in community psychiatric practice in the South African population. The use of a non-validated tool may have clinical implications. Tools with ordinal scales, such as the BPRS-E, are often used, and the scores are added to get a total. These total scores are then used to interpret symptom severity and guide clinical assessment and decision-making. If these tools are untested and their validity unknown for a specific population, all results obtained may not be valid. Consequently, clinical decision-making may be based on inaccurate and invalid information, resulting in missed symptomatology or overestimation of severity and thus incorrect and possibly harmful management decisions.

Therefore, this study intended to examine the construct validity of the BPRS-E when used in a South African community psychiatry setting using Rasch model analysis, thus evaluating if the items accurately assess the psychopathology in this population.

## Research methods and design

A quantitative analytic study design was employed. A retrospective review of clinical records with routinely measured and complete BPRS-E scores was conducted, and construct validity was evaluated using Rasch model analysis.

### Study setting

A Wits university-linked psychiatric service, staffed by three consultants, three registrars, two medical officers, one clinical psychologist and 20 psychiatric nurses, operates from nine public health care clinics in the district and serves approximately 4000 adults per month. People with a wide range of serious mental illnesses, including affective, psychotic, anxiety and substance use disorders, are referred to the service from primary health care, general hospital psychiatry wards and specialised psychiatric hospitals.^8^

### Study sample

The clinical records were purposively sampled at three of the Sedibeng community psychiatric clinics. The three clinics were chosen after discussion with the community psychiatric team as being the most suitable sites for the study. Clinical records were included if they were of adults aged 18 years and older in whom a BPRS-E was conducted as part of the routine clinical assessment between 01 January 2019 and 27 September 2019. Records were excluded if the BPRS-E was incomplete (items were missing), demographics were not documented or the psychiatric diagnosis had not been recorded clearly.

### Data collection and analysis

Data were collected between 01 October 2019 and 31 December 2019. Sociodemographic and clinical data and BPRS-E scores were collected. The BPRS-E scores were then analysed for construct validity using the Rasch model.

#### Rasch model analysis

According to Bond and Fox,^7^ Rasch model analysis is a theoretical mathematical description of how fundamental measurements are expected to work relating to psychological or latent variables. The Rasch model assesses whether results conform to strict expectations of scientific measurement. In other words, do the data fit the model close enough that the decisions we make are supported? The Rasch model allows measurements to be made from categorical data in the form of an adjustment between a respondent’s symptom severity (their ability) and the item difficulty.

Construct validity is also assessed by determining how well each item measures what it meant to measure. This is done by Rasch analysis by using fit statistics in which the expected versus observed responses are analysed. Overfit occurs when there is not enough variation, where more than one item measures the same thing, whereas underfit shows erratic variation which may indicate guessing. Misfit is an indication that the data are a good reflection of the sample.

#### Unidimensionality

Another aspect of the Rasch model that tests validity is unidimensionality, in which the model examines whether the question is measuring one single underlying construct. The principal component analysis of the residual values identifies the two most differing item subsets (i.e. the most negatively loading and most positively loading items). Then, to compare these two subsets, paired t-tests were performed. If less than 5% of the t-tests are significant, the scale is considered unidimensional, meaning that the items measure a single construct.^9,10^ If unidimensionality is achieved, the scores of the different items may be summed. The total score then represents the one underlying construct. This can be likened to factor analysis in that it assesses validity.

#### Differential item functioning

Carefully constructed tools can make good measurement estimates of symptom severity and thus may be useful; however, humans are complex, multidimensional and different. It is therefore important to consider that person-related factors, such as language comprehension, cultural understanding, education level and wording of questions, can reduce the meaningfulness of the answer provided by an individual. The Rasch model uses differential item functioning (DIF) to expose bias of items towards independent variables such as culture, education level, gender and the like.

Differential item functioning is a form of item bias that can occur when different patient groups within a sample, male versus female, despite having equal levels of the underlying trait (the same severity), have different responses for the same item. This means that items display different difficulty levels for different groups. For example, an item may favour females in that they identify with the question, whilst males may not. Differential item functioning was examined for each item with respect to patients’ sex and age and health care practitioner. The item estimates are examined graphically in the form of item characteristic curves (ICCs), which assume that the outcome of any interaction between the person and an item is determined by the person’s ability (illness severity) and the item difficulty (symptom severity). Uniform DIF occurs when the item response between groups with the same severity level shows a constant difference for all the items, whereas when the difference between groups is inconsistent, it is called non-uniform DIF.

### Ethical considerations

Permission to conduct research was obtained from Dr Victor Figueroa, the Sedibeng District Research Co-ordinator. Patient records were kept confidential throughout the study. Anonymity was preserved by assigning study numbers to individual records and utilising separate workbooks for the study numbers and data collection, with no links between the workbooks. The data collected had no identifying items. Ethical clearance was obtained from the University of the Witwatersrand Human Research Ethics Committee (No. M190911).

## Results

### Sample characteristics

A total of 192 individual patient records fulfilled inclusion criteria. The records were of adults ranging in age from 18 to 79 years (Table 1). Whilst male and female representation was approximately equal, males tended to be younger (median age 35 years) than females (median age 50 years). The BPRS-E was administered at a first visit to the clinic for 103 patients and at a follow-up visit for the other 89. Psychiatric nurses and doctors administered the BPRS-E in 75 and 117 patients, respectively.

**TABLE 1:** Sociodemographic characteristics of the sample.

Category	Sociodemographic variable	Number (*N* = 192)	Percentage
Age (years)	< 20	5	2.6
	20–29	41	21.4
	30–39	32	16.7
	40–49	40	20.8
	50–59	53	27.6
	60–69	17	8.9
	> 69	4	2.1
Gender	Male	93	48.4
	Female	99	51.6
Population group	Black African	152	79.2
	White	35	18.2
	Coloured	0	0.0
	Indian	5	2.6
	Other	0	0.0
Relationship status	Single	113	58.9
	In relationship	40	20.8
	Divorced or separated	19	9.9
	Widowed	20	10.4
Highest level of education	No education	11	5.7
	Primary	36	18.8
	Some secondary	70	36.5
	Matric	53	27.6
	Tertiary	22	11.5
Employment status	Employed	38	19.8
	Unemployed	143	74.5
	Studying or training	11	5.7
Disability grant status	Yes	30	15.6
	No	162	84.4

Working diagnoses in the clinical records were recorded using DSM-5 nomenclature. Schizophrenia spectrum and other psychotic disorders were the most frequent diagnoses, being made in just under a quarter of patients (Figure 1). The least represented diagnosis was that of somatic symptom and related disorders.

**FIGURE 1:**
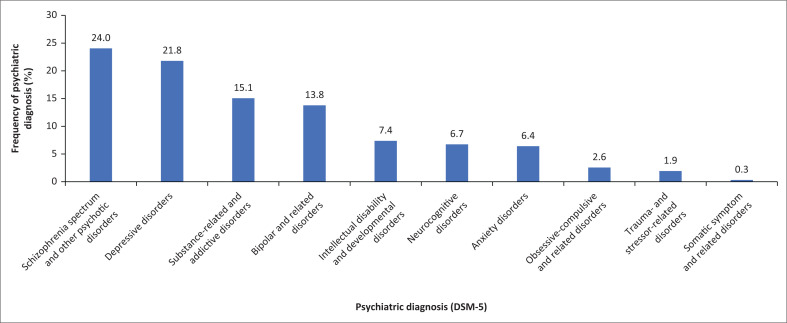
Psychiatric disorders.

### Brief Psychiatric Rating Scale – Expanded version scores

The frequencies of anchor points of scores 1–7 (not present, very mild, mild, moderate, moderately severe, severe and extremely severe) recorded for each item are presented in Figure 1-A1. Scores of 6 or 7 were most frequently recorded for depression (item 3) and anxiety (item 2) and not at all for uncooperativeness (item 20).

Total BPRS-E scores (i.e. the sum of the 24 items for each patient) ranged from 24 to 93 (Figure 2), with a median score of 38 and a mean of 41. As a score of 40 or more was recorded in just under half of the sample (*n* = 92; 48%), we used this cut-off to understand our sample population in terms of severity of psychopathology.

**FIGURE 2:**
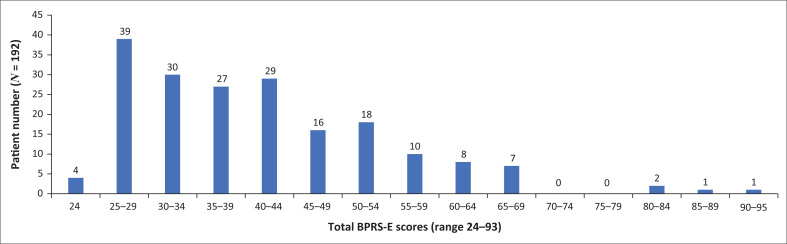
Total Brief Psychiatric Rating Scale – Expanded (BPRS-E) version scores for the sample.

Higher total scores were found more frequently in female compared to male patients, amongst those seen at a first visit rather than a follow-up visit, and when the BPRS-E was administered by a psychiatric nurse versus a doctor. In female patients, scores ranged from 25 to 93, with 56% (*n* = 55) scoring 40 or more, whereas in males, the scores ranged from 24 to 82, with only 40% (*n* = 37) scoring 40 or more. Of the 103 BPRS-E’s done at a first visit, 55% (*n* = 57) had a total score of 40 or more, whereas 39% (*n* = 35) of those done at a follow-up visit scored 40 or more. Total BPRS-E scores of 40 or more were found in just over half (53%, *n* = 40) of the 75 BPRS-Es administered by psychiatric nurses and in 44% (*n* = 52) of the 117 BPRS-Es administered by doctors. In terms of diagnosis, BPRS-E scores of 40 or more were found in the records of 53% of people with depressive disorders, 51% of those with bipolar disorder and 34% of those with schizophrenia spectrum disorders. The highest score of 93 was recorded for a person with an intellectual disability and a developmental disorder.

## Rasch model analysis

### Overall fit to the model

When using the original seven categories of severity (i.e. the anchor points from 1 to 7), all the threshold curves were disordered. However, when the categories were collapsed (as illustrated in Table 1-A1 for depression [item 3], elevated mood [item 7], hallucinations [item 10] and self-neglect [item 13]), the curves suggested a dichotomous (yes or no) or trichotomous (mild, moderate and severe) scale. This exercise (termed ‘rescoring’) was repeated for all items to enable a smooth transition from one severity level to another. The rescored anchor points for each item are presented in Table 2-A1 and Figure 2-A1. Of note, the severity categories were collapsed to a range of two to five categories, with the threshold maps remaining disordered for seven items after rescoring.

The initial analysis, using the recorded scores from the seven anchor points for each item labelled Run1, did not fit the requirements for a Rasch model analysis (Table 2). Whilst the chi-square value should be non-significant (*p* > 0.05) for fit to be achieved, it was 0.001479, indicating too much variance compared to that of the model. As the misfit in Run1 was partly because of the excessive disorganisation of the threshold curves, rescoring of the severity categories for all items increased the fit to the model (Rescore2). However, the improved fit in Rescore2 resulted in a loss in power of the analysis.

**TABLE 2:** Summary of Run1 and Rescore2 analysis.

Fit statistic	Ideal values	Run1	Rescore2
Chi-square probability (χ^2^)	Non-significant (> 0.05)	0.001479	0.742
Item mean fit residual	0	−0.445	−0.667
Person mean fit residual	0	−1.157	−0.519
Item standard deviation	1	1.058	0.762
Person standard deviation	1	0.907	0.8243
Reliability index/PSI	0.85	0.73	0.432
Coefficient alpha	0.8	0.806	0.706
Power of analysis of fit	Good or Excellent	GOOD	POOR†
Paired *t*-test analysis (Unidimensionality)	(PerC < 5%)	9.9%	4.69%

PSI, Person Separation Index.

Notwithstanding the overall misfit indicated by the chi-square value for Run1, the 24-item fit residuals (Table 3) in Run1 all fit within the ± 2.5 range, suggesting no overfitting or underfitting items and that each item contributed to a unique measure and correlated correctly. The item fit residual was maintained in Rescore2 for all items except for a slight underfitting of emotional withdrawal, indicating that the improved overall fit when the severity categories were collapsed did not significantly impact the contribution of each item to a unique measure.

**TABLE 3:** Item fit residuals – Run1 and Rescore2.

Item	Item description	Location		Fit residual		Chi-square	
		Run1	Rescore2	Run1	Rescore2	Run1	Rescore2
I0001	Somatic concern	−0.393	−0.618	2.450	0.576	12.188	0.216
I0002	Anxiety	−0.565	−1.368	0.033	−0.472	0.516	3.642
I0003	Depression	−0.682	−2.633	0.593	−0.130	1.039	1.271
I0004	Suicidality	−0.215	−0.526	0.699	−0.047	1.788	0.080
I0005	Guilt	0.018	0.019	−0.687	−0.373	4.469	1.232
I0006	Hostility	−0.276	−0.782	1.279	1.041	6.798	4.495
I0007	Elevated mood	−0.059	1.832	−0.966	−0.773	1.020	1.224
I0008	Grandiosity	0.094	−0.141	0.337	−0.388	1.551	0.591
I0009	Suspiciousness	−0.273	−0.075	−0.440	−0.527	0.824	0.671
I00010	Hallucinations	−0.412	−1.054	0.591	−0.340	6.369	1.841
I00011	Unusual thought content	−0.191	−0.300	−1.356	−1.162	2.201	2.328
I00012	Bizarre behaviour	−0.086	0.145	−0.990	−0.923	2.899	1.543
I00013	Self neglect	0.026	0.188	−1.148	−0.947	2.283	1.860
I00014	Disorientation	−0.143	0.206	−1.197	−0.490	3.482	3.650
I00015	Conceptual disorganisation	0.019	0.451	−1.527	−1.140	5.998	1.859
I00016	Blunted affect	−0.264	−2.030	−0.353	−2.143	0.345	1.234
I00017	Emotional withdrawal	−0.237	−1.882	−1.525	−2.544	6.603	3.494
I00018	Motor retardation	−0.106	−1.464	−0.539	−1.679	1.181	3.457
I00019	Tension	0.041	−1.103	−1.325	−1.148	5.881	1.919
I00020	Uncooperativeness	3.696	3.974	−0.798	−0.311	2.852	0.427
I00021	Excitement	0.054	1.811	−0.585	−0.559	1.595	1.198
I00022	Distractibility	−0.074	1.815	−1.818	−0.626	3.767	1.203
I00023	Motor hyperactivity	−0.064	1.815	−1.768	−0.626	2.770	1.203
I00024	Mannerisms and posturing	0.092	1.719	0.360	−0.282	4.142	0.740

### Unidimensionality

**FIGURE 3:**
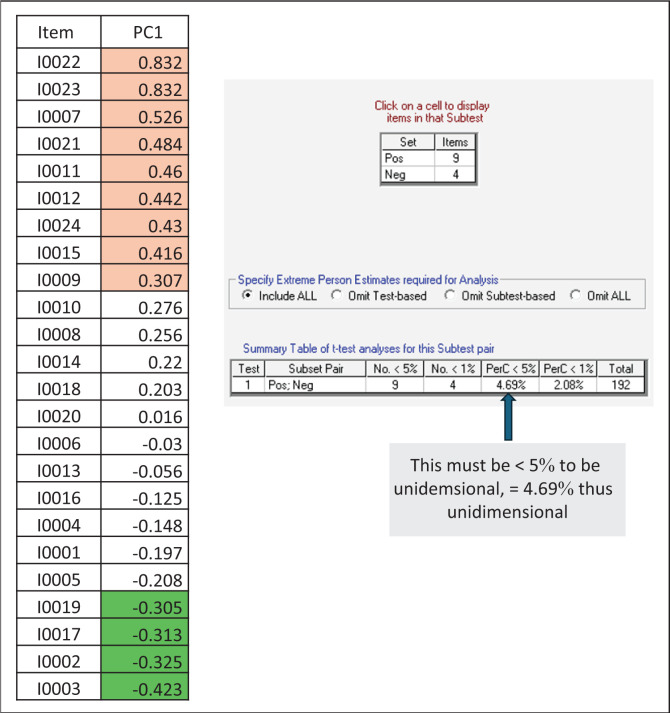
Principal component loadings Rescore2.

The principal component factor loadings of Rescore2 were analysed using positive and negative loading items. Unidimensionality was found for each item (Figure 3). The items highlighted in purple in Figure 3 showed the strongest positive loadings. This means that these items are positively correlated, and an increase in one is associated with an increase in the others. Items 22 (distractibility), 23 (motor hyperactivity), 7 (elevated mood) and 21 (excitement) were positively correlated and clustered in the clinical records of people diagnosed with bipolar and related disorders. Items 11 (unusual thought content), 12 (bizarre behaviour), 24 (mannerisms and posturing), 15 (conceptual disorganisation) and 9 (suspiciousness) also correlated and clustered for psychosis in the records of people diagnosed with psychotic disorders.

### Differential item functioning

**FIGURE 4:**
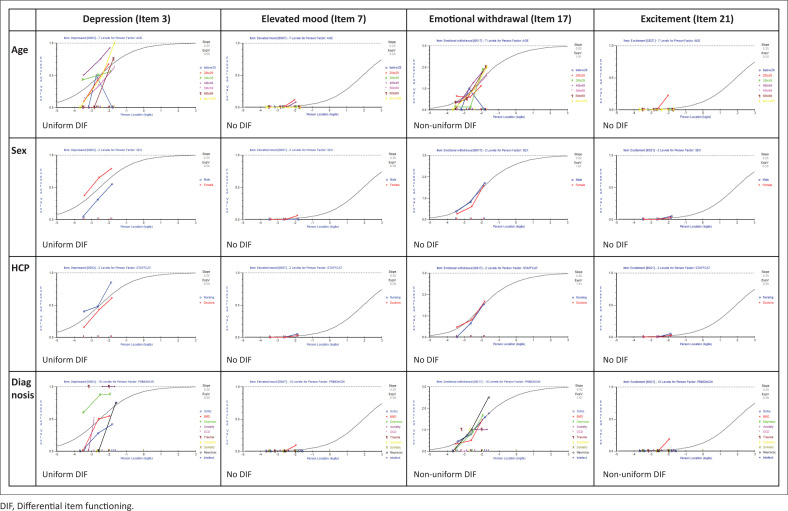
Differential item functioning for depression, elevated mood, emotional withdrawal and excitement.

The DIF was found for all the items and varied according to different items. Figure 4 illustrates the DIF results for items 3 (depression), 7 (elevated mood), 17 (emotional withdrawal) and 21 (excitement) when associated with age, sex, health care provider and diagnosis. Whilst uniform DIF was found for depression with each variable, no DIF was found for elevated mood, indicating consistent bias for depression but little to no item bias for elevated mood with respect to these variables. The non-uniform DIF found for emotional withdrawal with age and diagnosis, and for excitement with diagnosis, suggests erratic item bias with these particular variables.

## Discussion

In this study, we examined the construct validity of the BPRS-E when used routinely in a community psychiatric clinic using Rasch model analysis. For each of the 24 items, the analysis found good item fit and unidimensionality, indicating that each item measured the psychopathology it was intended to measure when used by mental health care nurses and doctors working in a South African community psychiatric setting. However, disordered thresholds for the 7-point severity scoring and inconsistent DIF were revealed, suggesting difficulty in applying the seven anchor points and differences in interpretation of some signs and symptoms with certain variables in the community psychiatric setting.

### Sample characteristics

The sample population’s demographics were similar in distribution to that of the general Sedibeng population in the 2011 census,^9^ and both demographics and clinical profile were comparable to the sample populations of both a cross-sectional, questionnaire-based study^6^ and a retrospective record review^11^ conducted in the Sedibeng District community psychiatry clinics.^8,11^ Therefore, although purposive sampling was used in our study, our sample population appears to reflect the Sedibeng District mental health care user population, implying that the BPRS-E was being administered amongst the full range of adult clinic attendees.

The use and interpretation of the BPRS-E scores, whether to aid decision-making for an individual or to evaluate the profile of patients attending the service, is dependent on the scale’s validity, the investigation of which was the purpose of this study.

### Construct validity

Two findings from the Rasch model analysis suggest good construct validity of the BPRS-E items when used routinely in the community psychiatric setting by different staff cadres. These were the item fit residuals and the test for unidimensionality of each item. Notably, each of the 24 items displayed a good fit to the Rasch model before collapsing the 7-point severity categories to improve the overall fit to the model. The individual validity of each item was confirmed in the test for unidimensionality, and the clustering of items for certain diagnoses was consistent with clinical expectations and factor analyses of the BPRS-E.^5^ Thus, the clinical assessment of the presence or absence of the different trait domains reported using the BPRS-E was consistent with modelled expectations, including in a population of mixed ethnicity in a peri-urban South African setting.

The construct validity of the BPRS-E is probably related to the flexibility of the tool in eliciting symptoms as well as the clinical experience of the psychiatric nurses and doctors in the Sedibeng District mental health service. Of importance, the tool provides examples of questions to ask for each symptom rather than prescribing set questions.^4^ The administrator is encouraged to enquire about symptoms in a manner that best elicits the trait in that individual and to incorporate all the information available, including collateral information, in making their assessment. This suggests that the BPRS-E allows the translation of Western medical descriptions into local ethnic understanding of psychopathology if the administrator has sufficient knowledge of both Western and local concepts of the different trait domains.

### Validity of the Brief Psychiatric Rating Scale – Expanded version scores

The disordered threshold curves and the need to collapse the severity categories to obtain overall fit to the model may indicate poor validity of the anchor points, and therefore the final BPRS-E scores, in a South African community psychiatric setting. The anchor points require careful assessment of the frequency, intensity and impact on functioning of each symptom and behaviour. Accurate assessment therefore demands precise enquiry, for which there may not be adequate time in our setting. In addition to the low staff:patient ratio,^8^ privacy and confidentiality may be compromised by a lack of infrastructure, as has been found in a study evaluating user experiences of integrated community psychiatry in the Sedibeng District.^11^

The disordered threshold curves may also be related to the small sample size and varied patient population. Noting that unstable results arise when individual items are analysed and that information is lost when the total score is used, Dazzi et al.^5^ suggested that subscales are used and derived four primary dimensions (positive symptoms, negative symptoms, affect and activation) from their meta-analysis of 32 BPRS-E studies. However, although two complementary meta-analytic methods were used, nine of the 24 items did not fit on any single dimension but loaded on multiple factors. It appears that when considering individual patients, each item would have value.

The temptation in our setting may be to reduce the severity scale from seven to four anchor points (none, mild, moderate and severe). However, such a reduction would reduce the sensitivity of the scale to change, confirmed to be useful in monitoring an individual’s response to care in an inpatient psychiatric unit.^12^ Collapsing severity categories may also compromise inter-rater reliability, as raters with varying clinical experience and training may have differing concepts of severity. An advantage of the BPRS-E is the precise description of each of the seven anchor points, allowing for objective assessment of severity of each item. The BPRS-E anchor points contrast with the lack of description of the Clinical Global Impression (CGI) scale, a 7-point scale whereby the clinician rates severity or improvement of illness according to an individual’s symptoms, behaviour and function with respect to the clinician’s overall experience with that patient population.^13^ Caution regarding inconsistent use of the CGI has been urged, noting that its psychometric properties have not been evaluated.^14^

In addition to adequate consultation time and suitable infrastructure, repeated training on the application of the anchor points is key. In their evaluation of the BPRS-E’s capacity to measure response to treatment, Burlingame et al.^12^ repeated their training of the researchers (all psychologists) every 4 months throughout the study to prevent ‘rater drift’. In our training, we possibly focussed on the clinical meaning of each item in terms of understanding psychopathology in our local context rather than on the anchor points.

Notwithstanding the Rasch model analysis results, our total scores were consistent with those found by Mapatwana et al.,^6^ who conducted a cross-sectional study amongst a sample of 120 patients at one of the Sedibeng District mental health clinics. The principal investigator (a psychiatrist fluent in the main local languages) administered the BPRS-E to all 120 patients in the study sample.

In our sample of clinical records, the total BPRS-E score ranged from 24 to 93, with a median of 38 and four patients recorded as having no symptoms. Although the BPRS-E scores reported by Mapatwana et al.^6^ (a range of 24–72, with a median of 30 and eight patients having no symptoms) were slightly lower than ours, they only included patients who had been deemed clinically stable at their last follow-up visit. In our study, the BPRS-E was administered at the patient’s first visit in over half (*n* = 103, 53.6%) of the sample.

Additionally, our finding of higher BPRS-E scores amongst female compared to male patients is consistent with the gender differences found in the South African Stress and Health Study.^15,16^ A nationally representative, interview-based study evaluating the prevalence of mental disorders in the general population, the South African Stress and Health Study, found an increased likelihood and greater severity of depression and anxiety disorders amongst female than male participants. Thus, in practice, the severity scoring conducted by the Sedibeng psychiatric nurses and doctors appears to be realistic.

### Clinical meaning of total Brief Psychiatric Rating Scale – Expanded version scores

We used a BPRS-E score cut-off of 40 or more to describe our sample population in terms of severity of psychopathology. Whilst this was an arbitrary cut-off, determined by the sample itself, there is no standardised mild, moderate or severe categorisation of the total scores.^5^ Referring to the 18-item BPRS, Leucht et al.^17^ noted that the clinical meaning of the total scores was uncertain and compared them to the CGI severity subscale in a patient population with schizophrenia. Although they established corresponding CGI scores for the BPRS, they concluded that the true value of the BPRS lay in the comparison of the scores over time within the same person.

More recently, Jones et al.^18^ validated a version of the CGI severity subscale for a mental health patient population in correctional services (CGI-C) against the BPRS-E. The two measures corresponded slightly differently for different patient populations. Clinical Global Impression-C scores of 3 (‘mildly ill’), 5 (‘markedly ill’) and 7 (‘extremely ill’) corresponded with BPRS-E total scores of 28, 39 and 52, respectively, amongst those with psychosis (*n* = 185); 30, 46 and 54, respectively, amongst those with mood or anxiety disorders (*n* = 290); and 29, 44 and 54 for the whole sample (*n* = 726). These figures suggest a lower threshold for an experienced clinician to consider a person with psychosis ‘markedly ill’ than someone with a mood or anxiety disorder. Therefore, whilst providing an objective assessment of severity, the BPRS-E score should be interpreted in the context of the psychopathology present.

Some of our scores indicate extreme illness in selected patients, a concern considering that these were all community-dwelling outpatients. However, this is not unrealistic in the South African mental health care setting, which is characterised by rapid deinstitutionalisation without the development of accessible care appropriate to the severity of the person’s condition.^19^ In Sedibeng District, the marked shortage of inpatient beds means that many people with severe illness may be cared for by family members or in care centres run by non-governmental organisations, together with the community psychiatric services.^10,11^

### Differential item functioning

The varied DIF found in the Rasch model analysis may indicate bias in the assessment of some items with certain variables. For example, the uniform DIF for depression with sex may reflect a difference in interpretation of depressive symptomatology in female and male patients, noting that there was no difference for elevated mood, emotional withdrawal or excitement with sex. Similarly, the uniform DIF for depression with a health care provider may increase greater sensitivity to depressive symptoms amongst nurses than doctors, or it could reflect that doctors are more likely to see a patient after they have been assessed by a nurse, and therefore some improvement in depression between visits.

The non-uniform DIF for emotional withdrawal and excitement with diagnosis is possibly related to the wider range of illness which may give rise to these behaviours. Importantly, the BPRS-E measures dimensions or traits, not disorders. Additionally, psychiatric diagnoses are often unstable, and the diagnoses recorded in our study are working clinical assessments, which may not always be accurate.

Considering the move towards a more dimensional approach to psychiatric assessment, the multidimensional nature of the BPRS-E is probably an advantage, as it provides a picture of the patient’s current condition regardless of diagnosis. Our study suggests that the BPRS-E may be incorporated into busy clinical assessments of patients with complex conditions. Whilst the speed at which it has to be administered may affect accuracy, the next person to see the patient may at least gain some insight into the patient’s presentation at a previous visit.

## Limitations

There are several limitations to the study. The completeness of the data was affected by poor record keeping, which resulted in many missing BPRS-E scoring sheets. The sample may not have been representative of other communities and may not be generalisable. Although this study was conducted within only one community, attempts were made to obtain a wide variety of cultural backgrounds and diversity via sampling records from township and suburban clinic settings. Even so there was a lack of diversity and potentially a lack of variation in the level of symptom severity (with most BPRS-E’s revealing low scores). The overall sample size was small, which may result in limited certainty in the findings. The study was a retrospective review, which limits the certainty in the correct application of the BPRS-E. Nevertheless, the study does confirm that the use of the BPRS-E is possible in the South African community psychiatric setting.

## Conclusion

This study shows that the BPRS-E is valid for use within a South African community setting, as shown by the Rasch analysis findings of good item fit and unidimensionality. However, the scoring categories require further investigation as there was inconsistent use of the 7-point Likert scale categories. This was revealed in the disordered thresholds that required condensation of categories and the DIF analyses showing significant bias in scoring between doctors and nurses for most items.

### Recommendations

This study revealed some interesting considerations regarding the BPRS-E in a South African community setting. Further research is needed to determine the extent of these implications. More training is required in the use of the BPRS-E, as well as frequent refresher sessions. There is a potential for adjustment of the tool to a South African context, utilising nurse input and buy-in to improve not only the quality of the implementation but the frequency. A further, possible recommendation is to review the scoring categories, and thus simplify and reduce the number of severity categories. Further research on the feasibility, acceptability and usefulness of the BPRS-E in the South African setting is recommended.

## Figures and Tables

**FIGURE 1-A1:**
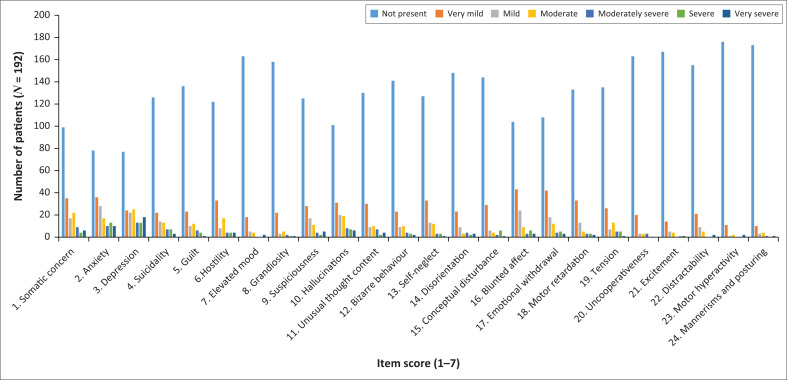
Frequency of anchor points or severity score by Brief Psychiatric Rating Scale – Expanded version items.

**FIGURE 2-A1:**
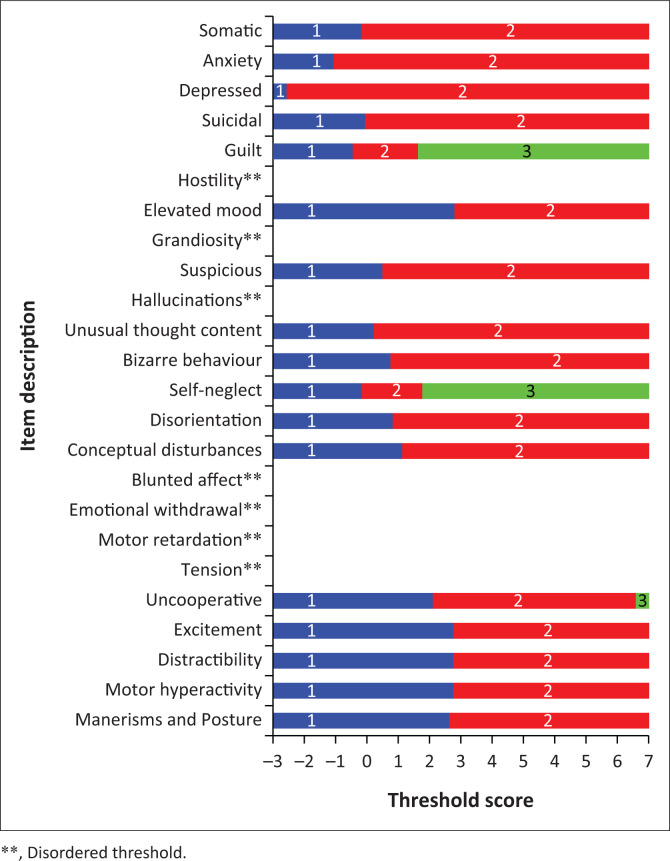
Threshold map Rescore 2.

## Appendix 1

**TABLE 1-A1:** Run 1 and Rescore 2 threshold maps for selected items.

Items	Item category curves – threshold maps	
	Run1	Rescore2
3. Depression		
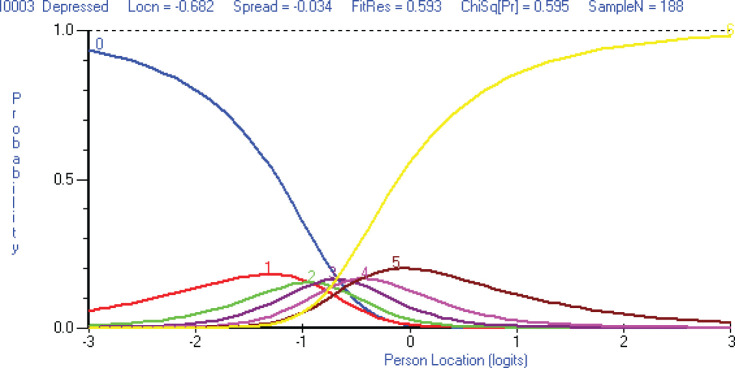
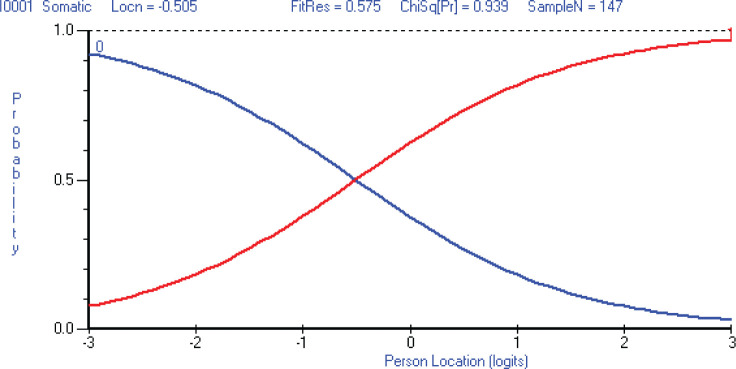
7. Elevated Mood		
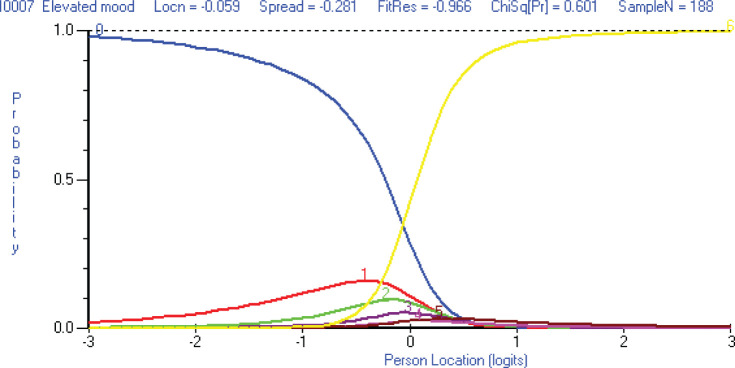
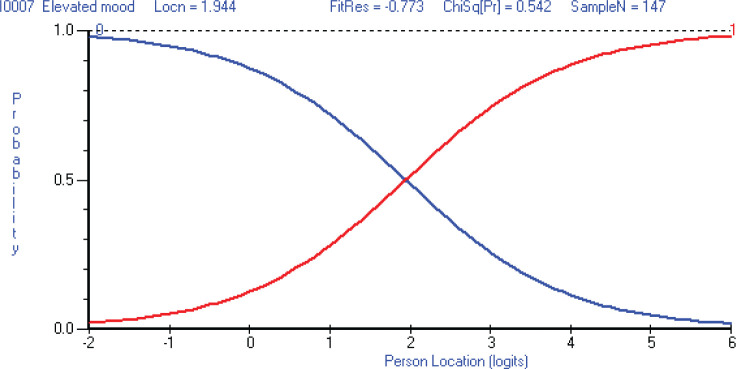
10. Hallucinations		
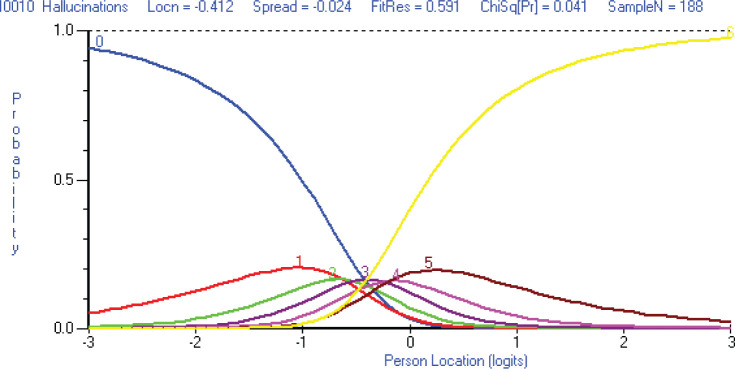
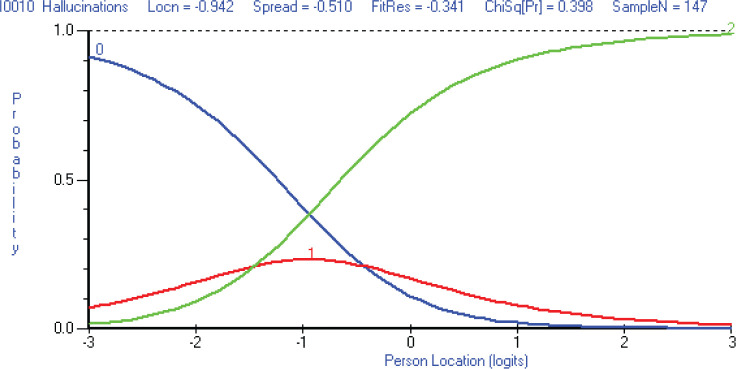
13. Self-neglect		
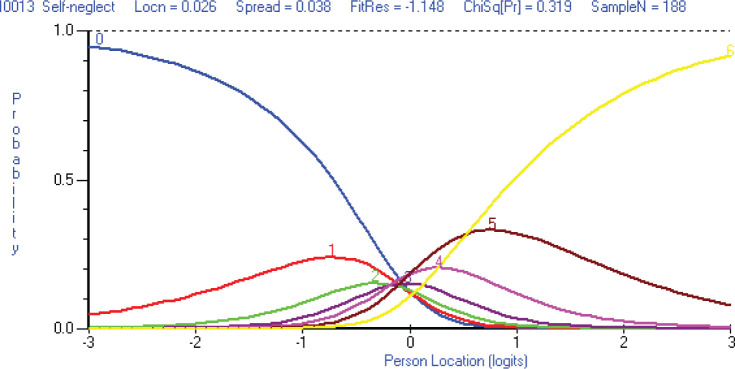
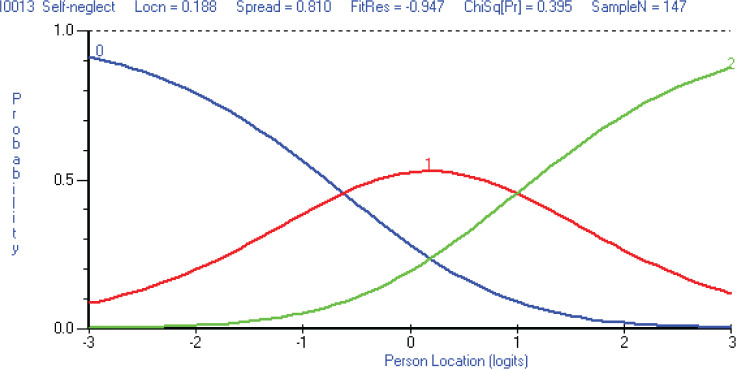

**TABLE 2-A1:** Rescoring of anchor points for each item (Rescore 2).

Item	Max score	Rescored	1	2	3	4	5	6	7
I0001	1	Y	0	0	0	0	1	1	1
I0002	1	Y	0	0	0	0	1	1	1
I0003	1	Y	0	0	0	1	1	1	1
I0004	1	Y	0	0	0	0	1	1	1
I0005	2	Y	0	0	0	1	1	1	2
I0006	2	Y	0	0	0	0	1	1	2
I0007	1	Y	0	0	0	0	0	1	1
I0008	2	Y	0	0	0	0	1	1	2
I0009	1	Y	0	0	0	0	1	1	1
I0010	2	Y	0	0	0	0	1	1	2
I0011	1	Y	0	0	0	0	1	1	1
I0012	1	Y	0	0	0	0	1	1	1
I0013	2	Y	0	0	0	1	1	1	2
I0014	1	Y	0	0	0	0	1	1	1
I0015	1	Y	0	0	0	0	0	1	1
I0016	4	Y	0	1	2	2	3	4	4
I0017	3	Y	0	1	2	2	2	3	3
I0018	4	Y	0	1	2	2	3	3	4
I0019	4	Y	0	0	0	1	2	3	4
I0020	2	Y	0	0	0	0	1	1	2
I0021	1	Y	0	0	0	0	1	1	1
I0022	1	Y	0	0	0	0	1	1	1
I0023	1	Y	0	0	0	0	1	1	1
I0024	1	Y	0	0	0	0	1	1	1
